# The Public Health Innovation Model: Merging Private Sector Processes with Public Health Strengths

**DOI:** 10.3389/fpubh.2017.00192

**Published:** 2017-08-07

**Authors:** Cameron Lister, Hannah Payne, Carl L. Hanson, Michael D. Barnes, Siena F. Davis, Todd Manwaring

**Affiliations:** ^1^Department of Health Science, Brigham Young University, Provo, UT, United States; ^2^Ballard Center for Economic Self-Reliance, Brigham Young University, Provo, UT, United States

**Keywords:** innovation, program planning, design thinking, leadership, private sector

## Abstract

Public health enjoyed a number of successes over the twentieth century. However, public health agencies have arguably been ill equipped to sustain these successes and address the complex threats we face today, including morbidity and mortality associated with persistent chronic diseases and emerging infectious diseases, in the context of flat funding and new and changing health care legislation. Transformational leaders, who are not afraid of taking risks to develop innovative approaches to combat present-day threats, are needed within public health agencies. We propose the Public Health Innovation Model (PHIM) as a tool for public health leaders who wish to integrate innovation into public health practice. This model merges traditional public health program planning models with innovation principles adapted from the private sector, including design thinking, seeking funding from private sector entities, and more strongly emphasizing program outcomes. We also discuss principles that leaders should consider adopting when transitioning to the PHIM, including cross-collaboration, community buy-in, human-centered assessment, autonomy and creativity, rapid experimentation and prototyping, and accountability to outcomes.

## Introduction

Recent achievements in public health have resulted in a 25-year increase in average life expectancy in the United States (1). These advances were the result of changes in the public health system, including improved surveillance systems, advocacy for effective health policies, and epidemiologic studies which improved decision-making capabilities (2). However, declining public health resources and complex health threats may make it difficult for advances of the past century to be sustained.

Public health frameworks have neither changed in response to such threats nor adapted in the face of technological and cultural shifts. For example, public health’s utilization of social media is inferior to fields such as business and marketing; while health departments have attempted to incorporate social media in practice, studies suggest that health professionals’ capacity for using these tools to engage populations is low (3–5). Indeed, it has been suggested that the current public health system has “neither the organization nor the incentive to comprehensively address population-centered, primary prevention health services that are evidence-based or linked to improved health outcomes” (6).

The inability, or reluctance, to adopt major advancements and reconstruct frameworks in public health may be attributed to the legacy concept. This concept is the tendency for a successful organization to believe it is entitled to continued success; as a result, the organization can fail to seek new opportunities, hampering continued success (7). Contemporary public health problems “require a different set of tools which will only be used if the legacy concept in public health is replaced by a new attitude that encourages innovation, risk-taking, and the building of new partnerships” (6).

To combat increasingly complex public health threats, public health leaders should pursue new processes and implement innovative solutions. In particular, traditional public health planning models do not explicitly encourage innovation. While the private sector conventionally resorts to innovative thinking, experimentation, and risk-taking in the face of threats, this approach is not yet embraced in public health’s program planning models. A new public health framework, which incorporates successful processes of the private sector and maximizes the strengths of the public sector, may be a major key to significant improvements in our most pressing and complex public health threats.

## Traditional Public Health Planning Models

**Figure 1:**
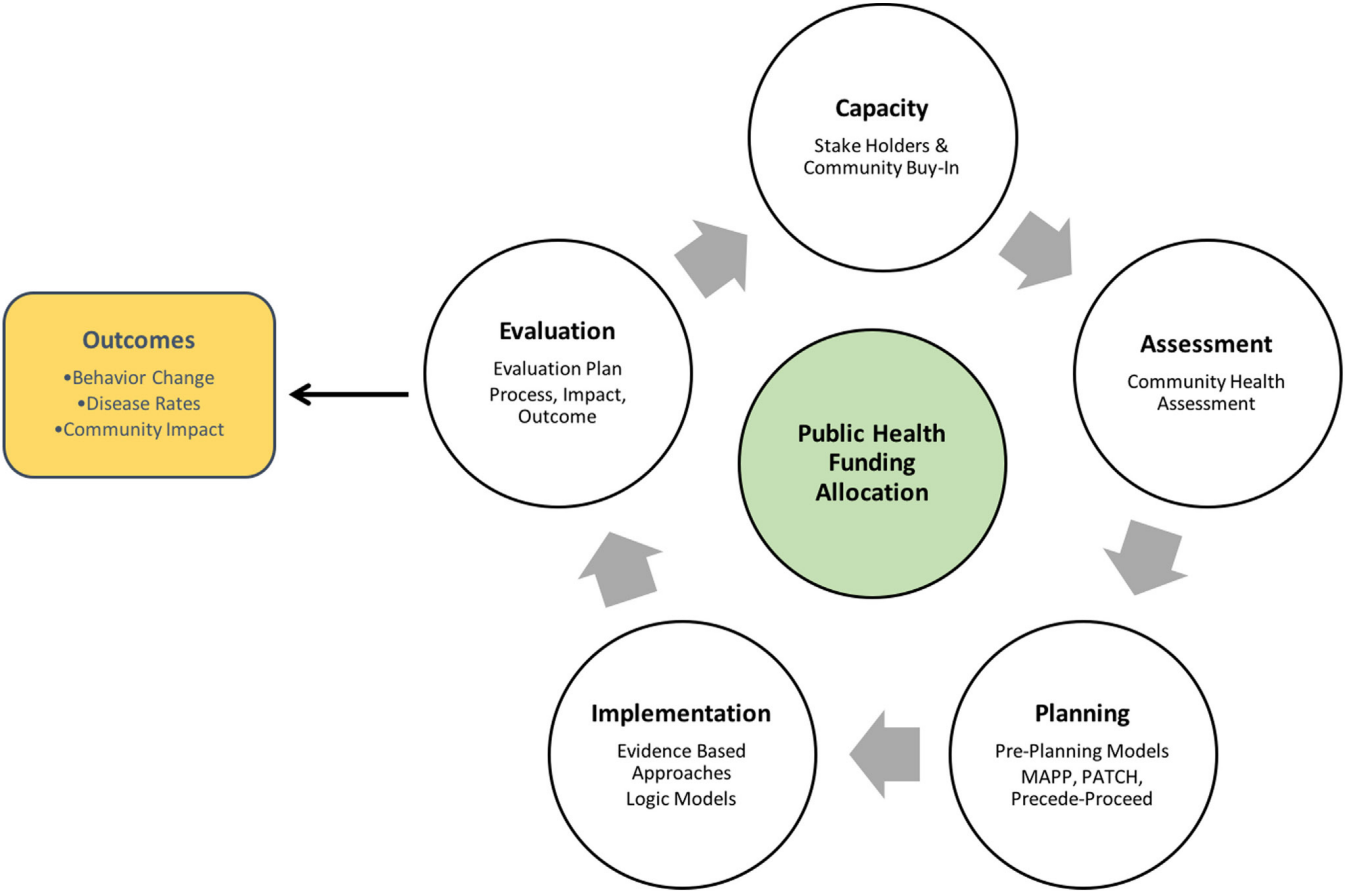
Traditional Public Health Planning Model [adapted from McKenzie et al. (9)].

In traditional public health planning models (see Figure 1), key characteristics are as follows:

1. Steps are linear, and solutions are often evidence based and preconceived before beginning the planning process. In these models, the goal of the health professional is not to generate novel solutions but to implement prescribed solutions in varying contexts.
2. Funding usually comes from government and public sources, such as the Prevention and Public Health Fund and state general funds (8). Public funding opportunities are often limited in availability and scope; consequently, practitioners may be constrained in the type and cost of programs they can implement.
3. Program outcomes are not strongly linked to funding allocation; while granting organizations do take into account program effectiveness, funding is usually allocated to organizations on a regular basis, independent of program outcomes.

Traditional public health planning models have been successful for many public health problems in the past. However, reliance on evidence-based practice and public funding, as well as neglecting to attend to program outcomes in allocating funding, can result in the de-emphasis of health outcomes in program implementation, and the long-term implementation of ineffective programs.

## Lessons from the Private Sector

### Innovative Solutions

**Figure 2:**
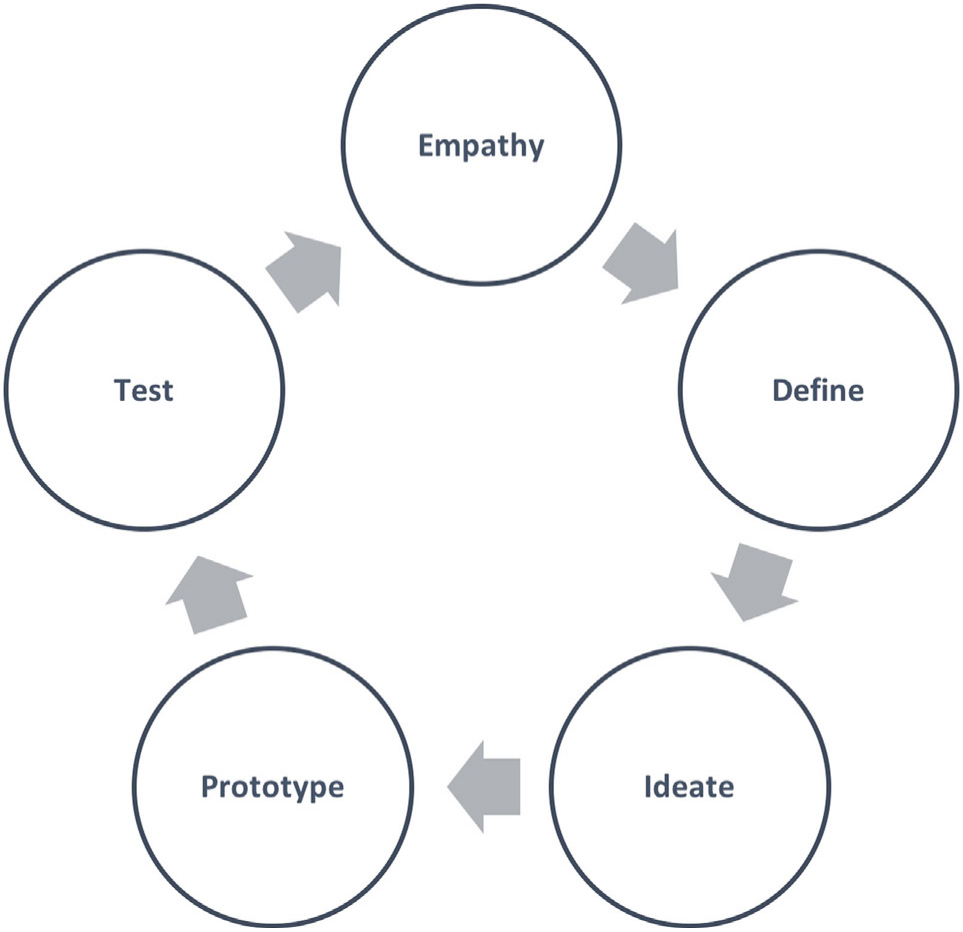
Design thinking process.

A new planning model must incorporate a mechanism for generating innovative solutions. Design thinking (see Figure 2), a problem-solving technique widely embraced in the private sector, is one such process. It is an approach to solving problems that starts with the customer and is human centered, research based, collaborative and multidisciplinary, and iterative (10).

Design thinking is said to find more innovative solutions to problems in less time and with less expense than traditional methods by initiating a continuous joint discovery cycle between the client and practitioner. Shortly after this process, an inexpensive, rapid prototyping cycle is initiated that creates “touchable” solutions that the client can test. These two interconnected cycles are repeated until a desirable and viable (as determined by the client) solution is found. Only then does the practitioner implement the new solution (10, 11).

While design thinking has been implemented by a number of fields in the private sector, it is less commonly utilized in the public sector, including in public health. Public health may be better able to incorporate innovation in practice with the adoption of principles from design thinking. Indeed, Trowbridge (12) suggests that “public health is well-positioned to expand application of design thinking to include health promotion.”

### Increase Funding from Private Entities

Distinct in a new model must be the addition of public health start-up funding from businesses and other private parties. Private funding is best allocated in the Prototype (or Implementation) mode (see Figure 2), in which practitioners propose and iterate small-scale program plans. Funders can then go on to implement larger scale iterations of the most promising ideas proposed by practitioners.

Seeking funding from public sources may be a challenging shift for health practitioners, as the leading health issues may not be the primary interests of private funders. Questions associated with balancing the power and interests, including conflicts of interests, are important to consider when partnering with private funders. Leveraging appropriate resources using important precautions can be taken to weigh benefits with any risks that could exist. Examples of well-cited precautions include at least three papers that note key tests to balance the power and interests of public–private partnerships while also promoting the benefits, minimizing the risks associated with leveraging increasingly sustainable partnerships in communities (13–15). These partnerships in public health already exist, including in health product development (16, 17) and the strengthening of health services (18). More clearly linking funding to outcomes may also be helpful to find better ways for valuating and monetizing prevention.

Public health practitioners must learn to procure additional funding from private entities as public health is scaled up to address an increasing variety of health needs among diverse populations. Public health practitioners can leverage the corporate social responsibility (CSR) component of private entities to advance public health programs. CSR has been defined by the European Commission as “a concept whereby companies decide voluntarily to contribute to a better society and a cleaner environment” (19). Examples of CSR include Ben and Jerry’s Caring Dairy program (a sustainability program for dairy farms) (20), Levi Strauss & Co’s Water‹Less™ process (which has saved one billion liters of water since 2011) (21), and TOMS One for One^®^ model (a model TOMS follows to provide shoes, sight, water, and safe birth services in return for every purchased product) (22).

Orlitzky et al. conducted a meta-analysis to understand the relationship between corporate social performance (CSP) and corporate financial performance (CFP) (23). Their findings suggest that there is a positive relationship between CSP and CFP. In other words, businesses that support social or environmental causes benefit though increased profits (23). In the context of public health, private organizations will likely provide continued funding to programs that help to fulfill their need for CSR and ultimately CFP.

## Public Health Innovation Model (PHIM)

**Figure 3:**
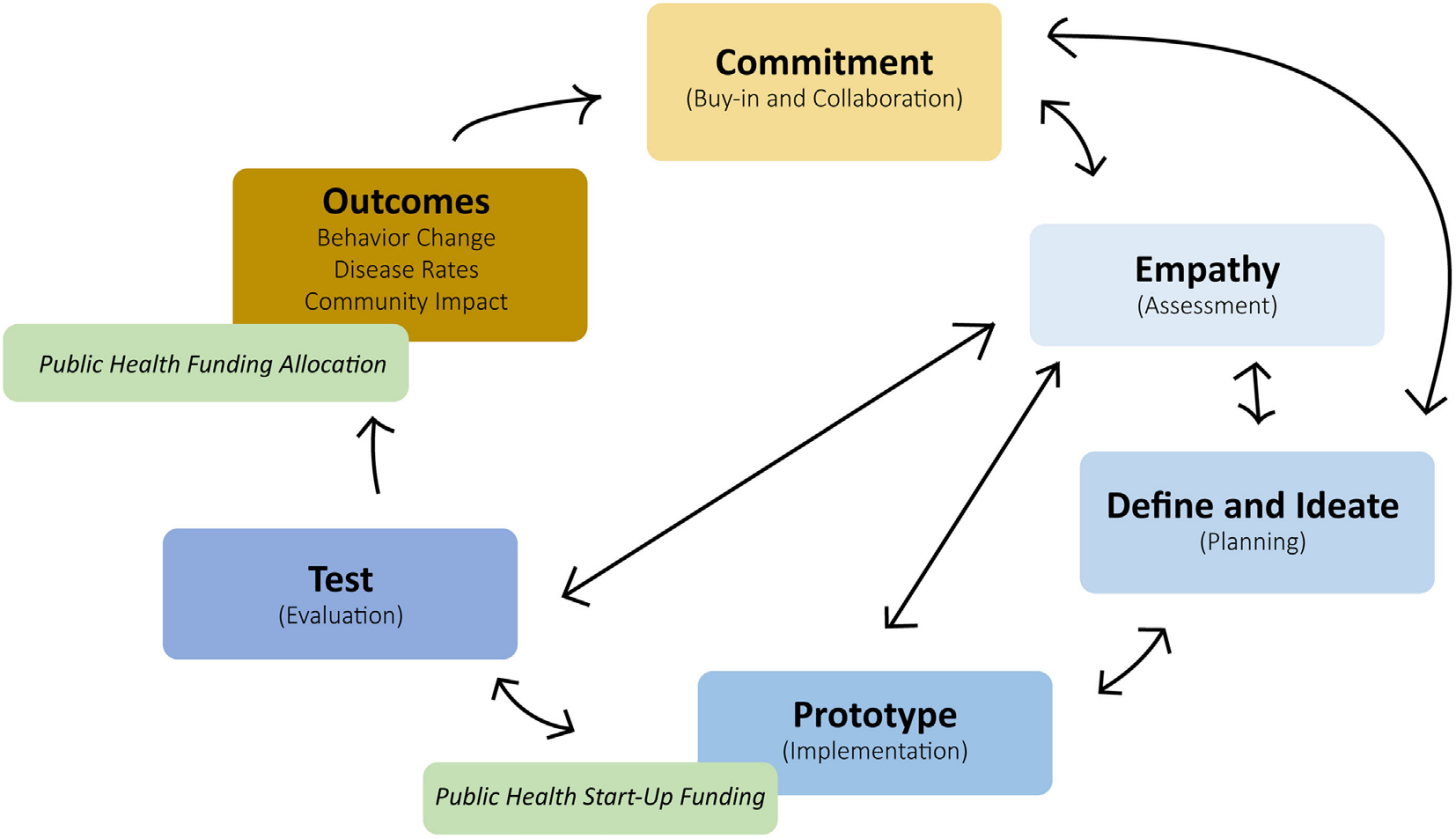
Public Health Innovation Model.

The PHIM merges traditional public health planning models with lessons learned from the private sector (see Figure 3). The PHIM accomplishes this by integrating design thinking “modes” with traditional program planning stages, leveraging the use of private sectors resources, and focusing more closely on program outcomes.

The successful adoption of private sector strengths into public health planning models requires public health to adopt several key strategies that include cross-collaboration, community buy-in, autonomy, and creativity. A discussion of these strategies and steps of the PHIM are discussed in more detail below. Examples of innovative public health approaches that incorporate these principles in practice can be found in Table 1.

**Table 1:** Examples illustrating Public Health Innovation Model (PHIM) and design thinking components.

Intervention or program	Program details	PHIM stage with activity highlights	Design thinking component
Peterborough Prison Bond (24–26)	Based on the idea of a Social Impact Bond, where the government only pays for a program if it meets a set of predetermined criteria of efficacy. Allows governments to try something new without the risk of embarrassment or failure	*Cross-collaboration and community buy-in*—Involves garnering support and buy-in from the private sector, who provides the initial capital	Empathy
		*Autonomy and Creativity*—A unique, novel program to reduce prison recidivism rates that encourages creativity and independent solutions that have never been done before to solve modern problems in modern ways	Define and ideate
	This program then partners with businessmen who are interested in investing in social change to reduce prison recidivism rates. They provide the initial capital for these programs and then receive payment from the government after their idea successfully reduces the problem (7–13% annual return on investment)	*Rapid Experimentation*—Because the threat of breaking a government contract is not present, individuals are able to change course rapidly to meet population needs	Prototype
		*Accountability*—The programs are held accountable to their outcomes; if they don’t prove they can solve the problem over time they are not funded	Test

Code for America—Adopt a Fire Hydrant (27)	This program involves “open sourcing the government,” or engaging citizens in caring for their communities from the ground up using new technologies	*Community Buy-In*—Solutions are not created unless regular citizens are not interested in and sold on ideas. People are organized through initiatives online and work toward common goals through donated time	Empathy
	People volunteer their time to create code to organize communities and solve social problems, such as coding an application that enables citizens to sign up to adopt fire hydrants near their residence	*Cross-Collaboration*—Organizations use resources and manpower from ordinary citizens to collaborate on social problems	Empathy
		*Autonomy and Creativity*—Adopt a Fire Hydrant uses twenty-first century methods and online technology to organize and facilitate social change. There is no bureaucracy or hierarchy, as innovations come from the bottom-up	Ideate
		*Rapid Experimentation and Prototyping*—Little bureaucracy involvement means individuals are able to select problems and solutions as they see fit and act accordingly; can very quickly alter solutions and try out new ideas	Prototype
		*Emphasis on Skills*—Individuals are not necessarily required to have experience in public health or social fields; rather, an emphasis on coding skills is required for citizens to create solutions	Prototype

Truth (28)	A rebranded version of the hard-hitting Truth campaign, this campaign targets millennials in an attempt to make this the last generation to smoke in the United States	*Cross-Collaboration and Community Buy-In*—The campaign requires buy-in and engagement from teens to be successful, as the success of the campaign requires teens to share material on social media	Empathy
		*Autonomy and Creativity*—This campaign uses social media, a widely available and twenty-first century platform, to spread awareness and prompt action toward eliminating smoking. The interactive and open-source nature of social media enables youth to contribute ideas and exercise independence and creativity to decrease smoking prevalence	Define and ideate
	Using irreverent and targeted social media, viral videos, and events aimed at youth, this antismoking campaign is one of the few awareness campaigns utilizing the full capacity of Web 2.0	*Emphasis on Skills*—Important skills emphasized in this campaign not necessarily standard in public health include social media, marketing, and humor, among others	Prototype
		*Rapid Experimentation*—Campaign managers can instantly measure success, reach, etc. of individual promotional materials through social media metrics, allowing them to tailor messages	Prototype

World Design Team (29)	A co-creation contest (ideation) used to generate novel concepts and ideas. Some companies seek innovation by requesting target participants to complete a given task within a given timeframe	*Community Buy-In*—The potential planning was built on participants dream-sheet ideas to formulate plans that could be easily tested or prepared for some form of experimentation	Empathy
	Boeing instituted a social media-based ideation to solicit 120,000 individuals around the world to be voluntary members of its World Design Team. Participants were invited to contribute their ideas to design a new 787 Dreamliner airplane	*Cross-Collaboration and Community-Buy-In*—Boeing used a forward-thinking question for innovation communicated through a virtual environment to foster creativity or innovation through the ideation strategy. In this case, social media allowed a broad group of participants whose ideas could emerge from everywhere in the world	Empathy and ideate
	This example illustrates how virtual environments can foster creativity or innovation through the ideation strategy		

This City is Going on a Diet (30)	Oklahoma City mayor, Mick Cornett was inspired to challenge his city to lose one million pounds following his own 42-pound weight loss. He partnered with fast food and local restaurants to promote healthy menu choices	*Cross-Collaboration and Community-Buy-In*—Was built by connecting various players together rather than blaming. Government leaders, retail merchants, and local citizens became united around the message that “you have to eat differently”	Empathy
	In early 2007, the city began the challenge as one of the fattest cities, and in early 2012, it had met its million-pound goal and also was listed among America’s top 10 fittest cities	*Start-Up Funding*—The mayor persuaded a healthcare magnate to fund the information website, and local news sources joined with their endorsements and support. Soon national media became champions of the initiative	Prototype
	The innovation took hold because it focused on food intake and not just exercise	*Community Testing*—By emphasizing an inclusive message, local participants tried different approaches with menu planning to help improve a culture of good choices	Test

### Commitment

In the capacity-building stage of traditional models (Figure 1), public health agencies develop informal partnerships with stakeholders to form decision-making teams. In the PHIM, the commitment stage incorporates two design thinking components (cross-collaboration and community buy-in) that can help to formalize partnerships over time:

*Cross-Collaboration*: Public health has recognized the importance of coalition building and interorganizational networks to not only improve health but also obtain resources and buy-in (31). However, the PHIM suggests that a stronger emphasis should be placed on the importance of cross-silo collaborations through the application of systems thinking. Incorporating systems thinking requires (1) attention to relationships and an understanding of people, (2) specialized study to understand the parts of the public health system, (3) transcending traditional academic boundaries, and (4) matching public health problems to the appropriate method for studying them (32). While it may not be possible to completely eliminate silos within public health, systems thinking has helped public health practitioners recognize that it is “essential to link [silos] … and recognize that they represent components of a larger system” (33).
*Community Buy-in*: Generating passion to solve community problems is a tenet of both design thinking and public health. When community members assemble together to tackle problems, the power of mobilization and local solutions begins to take place. Such grassroots efforts are typically more sustainable than top–down strategies employed by experts with little community involvement. Public health practitioners who are committed to community mobilization have learned to balance the use of best practice evidence while allowing for local innovation and creativity (34, 35). While community buy-in has been successfully implemented in recent health interventions (36, 37), successful long-term assessment is uncommon.

When approaching community buy-in using the PHIM, two principles can be adopted to increase success. First, public health can learn from businesses’ success in creating demand. Businesses typically achieve success by identifying a pain and then addressing that pain in such a way that the public becomes enthusiastic enough about the solution that they are willing to pay for it. Public health relies heavily on the free distribution of services, regardless of demand. Although this is unsurprising, as populations served are often economically disadvantaged, generating solutions to health pains similar to private organizations can be implemented by public health organizations. While there is much debate globally concerning the efficacy of charging for preventive health services, particularly in lower and middle income countries, in some cases, underserved populations may not view free services as valuable (38) and charging small, reasonable fees for health services may not negatively affect demand (39, 40). While there are limitations to charging for health services and instances in which this is inappropriate, creating the kind of demand typical in the private sector should be attempted more frequently.

Second, public health organizations should study and incorporate business models of innovation into practice, especially the Diffusion of Innovation Theory (41). This model seeks to explain how ideas gain momentum and diffuse through populations. The model achieves this by categorizing individuals into adoption stages (e.g., innovators, early adopters) and illustrating factors that influence the adoption of an innovation (e.g., relative advantage, compatibility) (41). Health practitioners can use this and similar models to design services and marketing efforts to increase appeal for targeted communities.

### Empathy and Assessment

In traditional models, after capacity building, health practitioners typically enter the Assessment stage, gathering data and input from the target population, often in the form of a community health assessment. The corresponding design thinking mode is the Empathy mode. This mode involves the “effort to understand the way [populations] … do things and why, their physical and emotional needs, how they think about the world, and what is meaningful to them” (10). While there is overlap between Empathy and Assessment, to better adopt the Empathy approach, public health practitioners may consider combining a human-centered approach with traditional assessment.

While data-driven approaches are crucial in community health assessments, a human-centered approach helps health practitioners to become more invested in the target population by promoting connection and more intimate interactions between health practitioners and those they serve. Such an approach can yield crucial insights into health problems that would not be possible with more formal approaches. Human-centered assessment may include more frequent face-to-face interactions with the target population, observing populations in their natural settings, approaching individuals with the intent to elicit stories as opposed to conduct interviews, and checking cultural biases.

### Define and Ideate

After assessment in traditional public health models is the Planning stage. Generally, this entails researching evidence-based programs and adapting such programs for the target population.

In design thinking, the Define and Ideate modes correspond with Planning. The Define mode entails defining the right challenge to address based on new understanding of populations; it is “an endeavor to synthesize … scattered findings into powerful insights” (10). Closely related is the Ideate mode, in which practitioners ideate potential solutions for the target population, often through brainstorming and other activities. In the Ideate mode, practitioners attempt to “step beyond obvious solutions,” “harness the collective perspectives and strengths of … teams,” “create fluency (volume) and flexibility (variety) in … innovation options,” and “get obvious solutions out of [team members’] … heads” (10). The Define and Ideate stage in the PHIM requires health practitioners to encourage autonomy and creativity in team members.

#### Autonomy and Creativity

Autonomy is a certain degree of freedom to test solutions and make decisions without fear of failure. The processes commonly used in the traditional public health planning model do not typically encourage creativity. First, public health professionals usually do not receive specific training to think creatively and innovatively. A reliance on evidence-based practice, while well meaning and useful in addressing familiar health challenges, is not appropriate when addressing the new or unfamiliar. Second, the evidence-based practice paradigm is generally based on the assumption that if a solution works in a handful of communities, it will work anywhere; more troubling still, many of the studies provided by organizations responsible for recommending evidence-based practice are out-of-date or infrequently updated.

To encourage health practitioners to act autonomously and creatively, program managers may consider encouraging workers to brainstorm ideas for the sake of generating insight into the problem at hand.

### Prototype

In the traditional public health model, Planning is followed by Implementation. The corresponding design thinking mode is Prototyping. Prototyping is “the iterative generation of artifacts intended to answer questions that get you closer to your final solution” and includes creating “low-resolution prototypes that are quick and cheap to make … but can elicit useful feedback from users and colleagues” (10).

In the Prototype mode, practitioners implement potential solutions with the goal to discover how they can improve their current model or program; in traditional Implementation, practitioners usually implement their programs full scale. The benefit of adopting the Prototype mode is that it allows health practitioners to better manage the solution-building process by breaking down problems and cheaply and quickly testing ideas (10). To adopt principles of the Prototype stage, public health practitioners must understand and implement the rapid experimentation and failure cycle characteristic of design thinking.

#### Rapid Experimentation and Failure

Rapid experimentation and failure are principles of success commonly found in the private sector, but not embraced in the current public health landscape due to limited funding opportunities. Intuit, a software company famous for their rapid experimentation framework, exemplifies the kinds of principles public health has the resources to implement on a microscale. At Intuit, employees are encouraged to generate innovative, even outrageous, ideas through building teams, gathering solutions, and creating and testing hypotheses. The key to Intuit’s success lies in employees’ ability to talk about ideas, test them quickly without spending exorbitant amounts of money, and have a healthy tolerance for failure (42).

In rapid prototyping, innovators iterate on theoretical and virtual prototypes until a “minimum awesome product” that “nails the pain” is created, as opposed to creating full-scale, error-free products that are expensive and require long development cycles (43). Despite differences between the products, audiences, and even motivations of the private and public sector, a mutually beneficial partnership between both sectors can develop on the basis of CSR. As stated previously, various private entities are motivated to engage in CSR for economic and ethical reasons. Public health practitioners can leverage the CSR component of the private sector for funds to initiate and sustain programs over time.

Furthermore, in public health, nailing the pain entails creating a health intervention or community plan that has enthusiastic buy-in from the community and is shown to change health outcomes. In traditional public health, such “prototyping” programs may take the form of pilot testing new ideas and conducting consumer research, but arguably, this is infrequently done in favor of evidence-based and traditional interventions, which are often required by granting organizations (44). Rapid prototyping allows for the testing of new ideas on a small-scale level and without extensive funding.

### Test and Outcomes

After program implementation, health practitioners move to the Evaluation stage, which usually includes program impact and outcome evaluations. The corresponding design thinking stage is the Test mode, in which practitioners solicit feedback about the prototypes they created previously to refine prototypes, learn more about their target population, and refine their problem statement. The end goal of Testing is to get closer to an ideal solution. Results from the Test mode often prompt practitioners to go back to the Empathize, Define, Ideate, and Prototype modes to refine solutions, which is distinct from traditional public health models.

Evaluation is one of the essential skills needed for innovation (45). In design thinking, outcome evaluation, or whether a program or prototype elicited a significant change in outcomes, is the most important method of determining whether a prototype or program was successful. While process and impact evaluations in public health can be useful in determining program success, focusing too much time on this type of assessment may detract resources from evaluations that most clearly demonstrate success. The end goal is to begin to make public health entities more accountable to the programs they produce. While public health is already concerned with program evaluation, the PHIM promotes dispersing the final funding allocation after outcomes have been assessed and programs have proven to be successful. These key structural changes to the funding structure emphasize the importance of achieving measurable outcomes and perpetuating programs that are successful, while eliminating programs that fail to make significant changes.

A common evaluation approach used with the traditional public health planning model is the use of logic modeling to demonstrate how inputs result in outcomes. Although innovation typically occurs through cross-collaboration, “a simple input-output or cause-and-effect model of evaluation is not appropriate.” (46) Newer, more sophisticated evaluation tools can be used when approaching evaluation from a systems thinking perspective. These tools can help with monitoring the interaction and connection between collaborators rather than simply the additive effects of inputs on outputs. Keane (47) has developed a tool to use “interactive” logic modeling to assess the impact of relationships (47). Capacity to conduct these types of evaluations will also continue to grow as more big data sources, such as electronic medical records, become available to public health.

## Conclusion

Innovation is not intended to replace public health best-practices or planning models but is available to enhance those practices and tailor interventions to meet local needs. Traditional public health planning models are useful, but practitioners are more likely to promote innovation by allowing opportunities for building commitment, empathy, ideation, and prototyping. Further, it is feasible that more challenging issues, such as persistent chronic or infectious diseases, can be better addressed through innovation-driven creativity and greater cooperation.

The aim of learning effective innovations can only come when there is a reasonable willingness to accept failures as essential for making improvements. The notion of “good failures” can be difficult for practitioners and stakeholders to accept because failure is often viewed as the antithesis of success. However, the key to good failure is that it can accelerate the learning process. The value of the PHIM is its ability to identify the hypothetical 1 strategy out of 10 that works. Evaluation of innovation requires a different perspective and should be viewed as a learning opportunity to identify what really works rather than implementing a well-intentioned approach that ultimately may not achieve an impact, which commonly occurs in practice today. Even when only 1 approach in 10 demonstrates success, that 1 approach can certainly help to inform future practice and lead to more impactful intervention.

## Author Contributions

All authors contributed toward idea formulation and writing.

## Conflict of Interest Statement

The authors declare that the research was conducted in the absence of any commercial or financial relationships that could be construed as a potential conflict of interest. HP and CL, former students at BYU, are now employees of Epic. The article was written while they were students in the MPH program at BYU. All other authors declare no competing interests.
